# Single-cell transcriptional profiling reveals the heterogeneity in embryonal rhabdomyosarcoma

**DOI:** 10.1097/MD.0000000000026775

**Published:** 2021-08-06

**Authors:** Bo Hong, Tian Xia, Chun-Jing Ye, Yong Zhan, Ran Yang, Jia Liu, Yi Li, Zhi-Xue Chen, Wei Yao, Kai Li, Jia Wang, Kui-Ran Dong, Rui Dong

**Affiliations:** a Department of Pediatric Surgery, Children's Hospital of Fudan University, and Shanghai Key Laboratory of Birth Defect, Shanghai, China; b Department of Orthopaedics, Children's Hospital of Fudan University, and Shanghai Key Laboratory of Birth Defect, Shanghai, China; c State Key Laboratory of Oncogenes and Related Genes, Renji-Med X Clinical Stem Cell Research Center, Ren Ji Hospital, School of Medicine, Shanghai Jiao Tong University, Shanghai, China.

**Keywords:** embryonal rhabdomyosarcoma, evolutionary history, GO/KEGG analysis, pseudo-time analysis, single-cell RNA sequencing

## Abstract

Rhabdomyosarcoma is the most common soft tissue sarcoma in children, and embryonal rhabdomyosarcoma is the most typical type of rhabdomyosarcoma. The heterogeneity, etiology, and origin of embryonal rhabdomyosarcoma remain unknown.

After obtaining the gene expression data of every cell in the tumor tissue by single-cell RNA sequencing, we used the Seurat package in R studio for quality control, analysis, and exploration of the data. All cells are divided into tumor cells and non-tumor cells, and we chose tumor cells by marker genes. Then, we repeated the process to cluster the tumor cells and divided the subgroups by their differentially expressed genes and gene ontology/Kyoto Encyclopedia of Genes and Genomes analysis. Additionally, Monocle 2 was used for pseudo-time analysis to obtain the evolution trajectory of cells in tumor tissues.

Tumor cells were divided into 5 subgroups according to their functions, which were characterized by high proliferation, sensing and adaptation to oxygen availability, enhanced epigenetic modification, enhanced nucleoside phosphonic acid metabolism, and ossification. Evolution trajectory of cells in tumor tissues is obtained.

We used pseudo-time analysis to distinguish between mesenchymal stem cells and fibroblasts, proved that embryonal rhabdomyosarcoma in the pelvic originated from skeletal muscle progenitor cells, showed the evolutionary trajectory of embryonal rhabdomyosarcoma, and improved the method of evaluating the degree of malignancy of embryonal rhabdomyosarcoma.

## 1 Introduction

Rhabdomyosarcoma is the third most prevalent extracranial solid tumor in childhood (about 6/1,000,000), accounting for approximately 4.5% of all childhood cancer cases.^[1–3]^ There are 4 clinical subtypes of rhabdomyosarcoma; among them, embryonal rhabdomyosarcoma (ERM) is the most common subtype (about 75%).^[4]^ The origin of ERM is unclear, but it is currently thought to originate from skeletal muscle progenitor cells (SMPCs) or endothelial progenitor cells (EPCs), which may be related to the distribution of ERM.^[2,3]^*PAX7* gene expression is an essential marker of skeletal muscle differentiation,^[5]^ and *RAS* gene family are abnormally expressed in ERM.^[6]^ They are of particular significance in the diagnosis of ERM.

Single-cell RNA sequencing (scRNA-seq) is a type of sequencing technology that obtains the complete gene expression information of every cell. This method can fully define the expression of transcription factors, growth factors, receptors, solute transporters, and other proteins in every cell, and it has been used in tumors to study tumor heterogeneity.^[7]^ Compared with traditional DNA sequencing methods, scRNA-seq avoids interference from background cells and captures important information expressed within only a few cells, such as the mutation status of tumor cells, the epigenetic status, and the expression level of related proteins.

In this study, we used scRNA-seq to reveal the heterogeneity in ERM and explore the evolutionary history of ERM.

## 2 Methods

### 2.1 Patient and tumor tissue

This study included a patient with ERM in the pelvic region who underwent imaging (computed tomography and magnetic resonance imaging) and immunohistochemistry, and the patient was a 6-year-old boy with ERM in stage III. This study was approved by the ethics committee of Fudan University Children's Hospital and informed consent was obtained from the participant's guardian. All experiments were carried out in accordance with the relevant guidelines and regulations.

### 2.2 Single-cell separation

The tumor tissue was cut into small pieces; collagenase IV (Gibco) and DNase I (Sigma) were added and the mixture was stirred at 37°C for 30 min. The samples were filtered through a 70-μM cell filter; the filtrate was washed in phosphate-buffered saline with 1% bovine serum albumin and 2 mM ethylenediaminetetraacetic acid and centrifuged at 500×*g* for 8 min. The single-cell suspension was separated by human lymphocyte separation fluid (CL5020; Cedarlane), and red blood cells and cell debris were removed according to the manufacturer's specifications. The granulosa cells were resuspended in phosphate-buffered saline with 1% bovine serum albumin, and their activity and size were evaluated with a Countess II FL instrument (Thermo).

### 2.3 scRNA-seq library preparation, and RNA-seq

ScRNA-seq libraries were prepared according to the Single Cell 3’Reagent Kit User Guide v2 (10× Genomics).^[8]^ In short, cellular suspensions were loaded on a Chromium Controller instrument (10× Genomics) to generate single-cell gel bead-in-emulsions (GEMs). After the formation of GEMs, the cells were lysed, and the GEMs were automatically dissolved to release several barcode sequences. mRNAs were reverse-transcribed to generate cDNAs with barcode and UMI information, and a cDNA library was established. The library was then pooled and sequenced on a NovaSeq 6000 (Illumina) at a depth of approximately 400M per sample. Raw sequencing data were converted to FASTQ files with Illumina bcl2fastq (version 2.19.1) and aligned to the human genome reference sequence (GRCh38). CellRanger (10× Genomics, version 2.1.1) was used to perform data processing to filter out the barcodes associated with low-quality cell bar codes.^[9]^

### 2.4 Seurat analysis and GO/KEGG analysis

Data were imported into R Studio (R 3.6.2), and the Seurat package (Seurat 3.1.4, https://github.com/satijalab/seurat) was used to process and analyze the obtained gene expression data. From quality control, genes expressed in no more than 3 cells were filtered out, and cells with more than 500 genes and less than 10% mitochondrial genes were selected. Then, the remaining data were standardized and normalized. After quality control and normalization, the nonlinear dimension reduction algorithm principle component analysis was implemented. Finally, cluster analysis was used to identify cell subtypes and Uniform Manifold Approximation and Projection for visualization of dimension reduction results. A heatmap of the top 10 genes unique to every cluster was displayed according to the log2FC values.

FindAllMarkers function was used to export the differentially expressed genes (DEGs) of every cluster during Seurat analysis. After Seurat analysis, we used the clusterProfiler package (https://bioconductor.org/packages/clusterProfiler) to perform gene ontology (GO)/Kyoto Encyclopedia of Genes and Genomes (KEGG) analysis. First, the bitr function was used to convert the gene ID. Then, the enrichGO and enrichKEGG functions were used to perform GO/KEGG enrichment analysis of the DEGs, and GO/KEGG terms with false discover rate less than 0.01 or 0.05 were considered significantly enriched. Finally, the enrichplot package was used to visualize the enrichment results.^[10]^

### 2.5 Pseudo-time analysis

We used Monocle 2 (http://cole-trapnell-lab.github.io/monocle-release) to conduct pseudo-time analysis in the cells. Monocle 2 used an algorithm to learn the changes in gene expression sequences that each cell must go through as part of a dynamic biological process (differentiation, for example). The cells were reduced dimensionality by the DDRTree method, sequenced in pseudo time, and finally visualized.^[11]^

### 2.6 Cluster cell-type annotation

We used the feature plot function to highlight the expression of known marker genes to identify clusters, and GO/KEGG analysis was performed on DEGs of every cluster to verify the correctness of the marker genes we selected. Parts of the cluster could not find marker genes, GO/KEGG analysis of DEGs in the clusters.

## 3 Results

### 3.1 The cellular composition of ERM tumor tissue obtained by Seurat analysis

**Figure 1:**
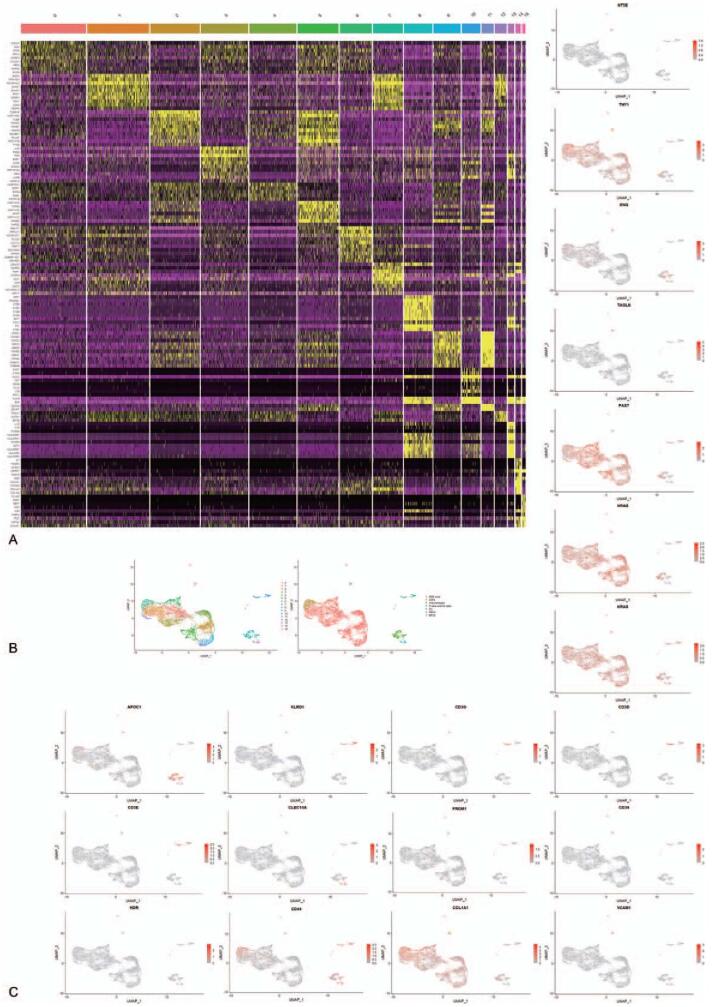
Seurat analysis results of tumor tissue. (A) Heatmap of the top 10 marker genes for every cluster. Yellow indicates high expression of a particular gene, and purple indicates low expression. (B) Cells are clustered and definitions of cell types are displayed in the uniform manifold approximation and projection (UMAP) plot. Every cluster is named. (C) Feature plots of marker genes in every cluster.

Seurat analysis was performed in all cells in the tumor tissue, and the heatmap of the top 10 marker genes for every cluster was shown in Figure 1A. The cellular composition of tumor tissues was defined by marker genes. The *APOC1* gene was activated when monocytes differentiate into macrophages,^[12]^ so cluster 8 comprised macrophages. *CD3* is a co-receptor on the surface of T cells that is expressed at all stages of T cell development and is a classic marker of T cells.^[13]^*CD94*, a *KLRD1* gene encoding product, acts as a receptor for NK cells and some cytotoxic T cells to recognize *MHC-1* (*HLA-E*).^[14]^ Therefore, cluster 10 was composed of T cells (*CD3*+)^[15]^ and NK cells (*CD3*−).^[16]^*CLEC10A* is a specific marker of human *CD1C*+ dendritic cells,^[17]^ so cluster 13 comprised dendritic cells. *CD34*, *PROM1*, and *KDR* are common marker genes of EPCs,^[18]^ so cluster 15 comprised EPCs. Cluster 7 can be labeled with *CD44* and *COL1A1*. *CD44* is a marker gene of mesenchymal stem cells (MSCs) and *COL1A1* could be expressed in fibroblasts,^[19,20]^ so cluster 7 might comprise MSCs or fibroblasts (hereinafter, undefined cells-1). Cluster 14 could be labeled with *CD44*, *VCAM1*, and *TAGLN* genes, which are expressed in both MSCs and fibroblasts so that Cluster14 may also comprise MSCs or fibroblasts (hereinafter, undefined cells-2).^[19–24]^ In Section 3.3.2, we knew cluster 7 comprised cancer-associated fibroblasts (CAFs) and cluster 14 consisted of MSCs. The remaining undefined cells were tumor cells and could be defined by *PAX7*, *HRAS*, and *KRAS* genes.^[5,6]^ The plot of ERM tumor tissue cellular composition was shown in Figure 1B, and feature plots of the above marker genes were shown in Figure 1C.

### 3.2 The heterogeneity in ERM tumor cells obtained by Seurat analysis and GO/KEGG analysis

**Figure 2:**
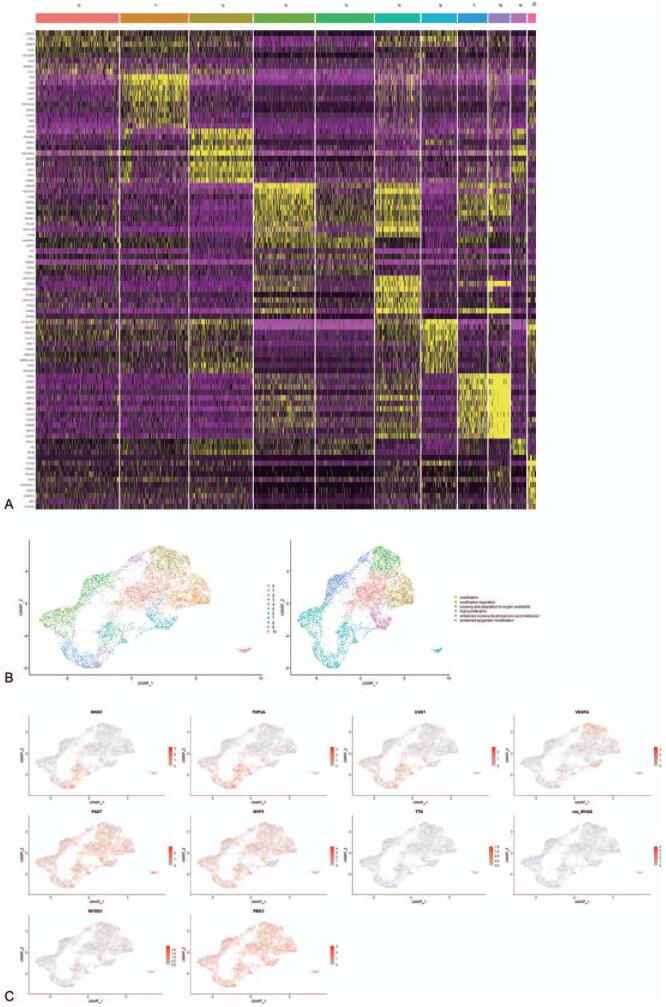
Seurat analysis verify heterogeneity in ERM. (A) Heatmap of the top 10 marker genes for every cluster. (B) Cells are clustered and definitions of cell types are displayed in the UMAP plot. Every cluster is named. (C) Feature plots of marker genes in every cluster. ERM = embryonal rhabdomyosarcoma.

To investigate the heterogeneity of tumor cells in ERM, we extracted tumor cells. Then, Seurat analysis and GO/KEGG analysis was performed for them. The heatmap of the top 10 marker genes for every cluster was shown in Figure 2A, and the plot of ERM tumor cell subgroups was shown in Figure 2B.

The first subgroup of cells (clusters 3, 5, 7, and 8) was highly proliferative and was labeled with *MKI67*,^[25]^*TOP2A*,^[26]^ and *CDK1* genes.^[27]^ According to GO and KEGG analysis of the DEGs in every cluster, this group of cells could be further divided into 2 subgroups. Cluster 3 was in G1 and S stages of mitosis, and the DEGs were mainly involved in the synthesis of DNA, RNA, and chromosomes, such as deoxyribonucleoside triphosphate metabolic process, DNA biosynthetic process, etc. Clusters 5, 7, and 8 were in G2 and M stages of mitosis, and the DEGs were mainly involved in the mitotic nuclear division, organelle fission, chromosome segregation, and sister chromatid segregation. Although cluster 10 was also highly proliferative, it was different from clusters 3, 5, 7, and 8. Marker genes for high proliferation could not define cluster 10, and the GO/KEGG analysis results in cluster 10 were different from other clusters too. GO/KEGG analysis showed that cluster 10 genes were mainly involved in the positive regulation of cell cycle, regulation of mitotic cell cycle phase transition, and regulation of cell cycle phase transition. The second subgroup of cells (cluster 2) could sense and adapt to oxygen availability and promote angiogenesis; this group was labeled with the *VEGFA* gene. GO/KEGG analysis showed that the DEGs in this group were mainly involved in response to hypoxia, decreased oxygen levels, and *HIF-1* signaling pathway. The metabolism of nucleoside phosphoric acid was enhanced in the third subgroup of cells (clusters 4 and 9). GO/KEGG analysis showed that the DEGs were mainly involved in the metabolism of nucleoside phosphoric acid, ATP metabolic process, and oxidative phosphorylation. The epigenetic modification was enhanced in the fourth subgroup of cells (cluster 6). GO and KEGG analysis showed that the DEGs in this group of cells were mainly involved in protein modification, RNA splicing, chromosome tissue regulation, and other epigenetic modification processes. The fifth subgroup of cells (clusters 0 and 1) may have ossification and ossification regulation functions. GO and KEGG analysis showed that DEGs in cluster 0 were mainly involved in ossification, and those in cluster 6 were mainly involved in ossification regulation. The feature plots of the above marker genes were shown in Figure 2C, and GO analysis results were shown in Figure S1, Supplemental Digital Content.

### 3.3 Pseudo-time analysis in cells

#### 3.3.1 The evolutionary trajectory of undefined cells

**Figure 3:**
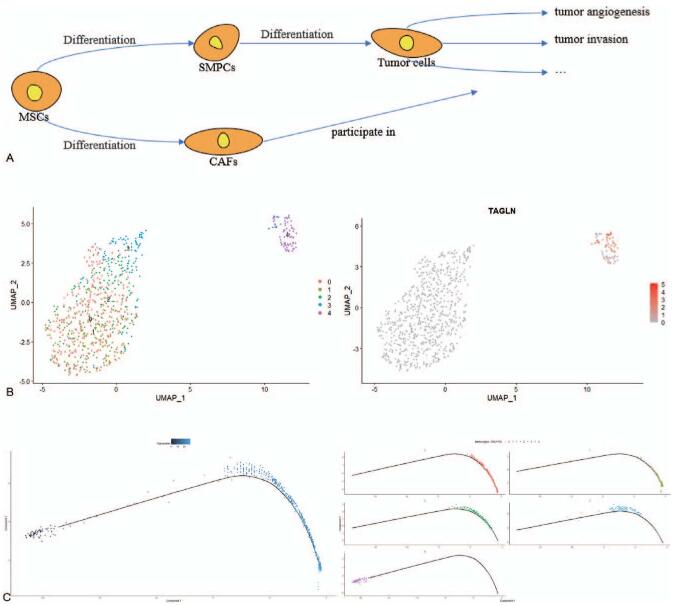
Pseudo-time analysis in undefined cells. (A) Evolutionary history of ERM. (B) Clustering cells and definitions of cell types in ERM are displayed in the UMAP plot. (C) Pseudotime trajectory of undefined cells, and dark blue is the start of pseudo time. ERM = embryonal rhabdomyosarcoma.

Undefined cells were extracted and regrouped, and Seurat analysis was performed to divide the cells into 5 clusters (Fig. 3B). Among these clusters, cluster 4 comprised undefined cells-2 (*TAGLN*^hi^, Fig. 3B), and the remaining clusters comprised undefined cells-1. The results of the pseudo-time analysis showed that the left branch was the beginning of the evolutionary trajectory. Undefined cells-1 were mainly located on the right side of the evolutionary trajectory and undefined cells-2 were mainly located on the left side of the evolutionary trajectory (Fig. 3C).

#### 3.3.2 The evolutionary trajectory of tumor cells, EPCs, and undefined cells

**Figure 4:**
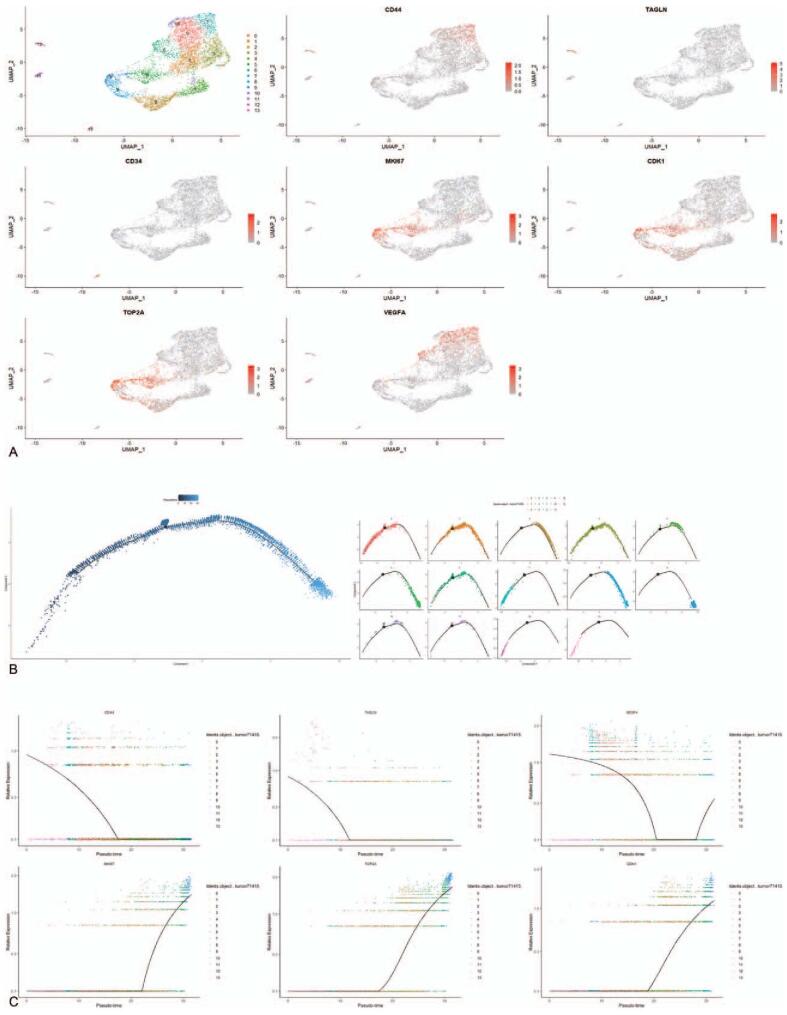
Pseudo-time analysis in tumor cells, EPCs, and undefined cells. (A) Clustering cells and definitions of cell types in ERM are displayed in the UMAP plot, and feature plots of marker genes in every cluster. (B) Pseudotime trajectory of the cells. (C) Trendgrams of highly expressed genes in the cell subtypes during pseudo time. EPCs = endothelial progenitor cells.

Tumor cells, EPCs, undefined cells were extracted and regrouped, and Seurat analysis was performed to divide the cells into 14 clusters (Fig. 4A). Cluster 7 comprised undefined cells-1 (*CD44*^hi^*TAGLN*-), cluster 12 comprised undefined cells-2 (*TAGLN*^hi^), cluster 13 comprised EPCs (*CD34*^hi^) (Fig. 4A), and the remaining clusters were composed of tumor cells. Pseudo-time analysis results showed that clusters 7, 12, and 13 were mainly located at different positions of the left branch of the evolutionary trajectory (Fig. 4B).

## 4 Discussion

The cellular composition of ERM tumor tissue is complex, and we have not yet distinguished whether undefined cells are mesenchymal stem cells or fibroblasts in Section 3.1.

According to minimal criteria for defining MSCs of the international society for cellular therapy position statement, MSC must express *CD105*, *CD73*, and *CD90* surface molecules.^[28]^*CD73* and *CD105* are not expressed in undefined cells-1, so undefined cells-1 are not composed of MSCs but fibroblasts (Fig. 1C). *VEGFA* is a member of the *PDGF*/*VEGF* growth factor family that plays an essential role in tumor angiogenesis^[29]^ and is expressed in undefined cells-1. Angiogenesis can provide oxygen and other nutrients for tumor invasion.^[30]^ Additionally, the generation of new blood vessels also provides a pathway for tumor invasion, along which tumor cells can invade the vascular system.^[31]^ In summary, undefined cells-1, like fibroblasts, participates in important aspects of solid tumor biology, such as tumor invasion, angiogenesis, and metastasis, which can be identified as CAFs.^[32]^

As for undefined cells-2, they meet minimal criteria for defining MSCs (Fig. 1C); thus, it cannot be denied that they are composed of MSCs. Pseudo-time analysis results (Fig. 3C) show that CAFs are mainly located at the right side of the evolutionary trajectory and undefined cells-2 are mainly located on the left side of the evolutionary trajectory, so undefined cells-2 appear earlier in the development process and it can be considered that CAFs originate from undefined cells-2. In this study, CAFs may originate from MSCs differentiation or activation of resident fibroblasts. If CAFs arise from activation of resident tissue fibroblasts via signals from tumor cells, tumor cells should appear earlier in the process of tumorigenesis and are closer to the starting point of the evolutionary trajectory.^[33]^ However, CAFs and tumor cells begin to appear in the same position along the evolutionary trajectory (Fig. 3C). Therefore, CAFs originate from MSCs rather than resident fibroblasts, and undefined cells-2 comprise MSCs.

After identifying the cellular composition of ERM tumor tissue, we begin to explore the evolutionary history of ERM.

*PAX7* and *MYF5* (marker genes of satellite myogenic cells and myoblasts) are expressed in tumor cells, but *MYOG*, *MYOD1*, and *TTN* (marker genes of myocytes) are rarely expressed in tumor cells (Fig. 2C).^[34]^ Therefore, ERM is regarded as an arrested state in normal skeletal muscle development and is hence considered to originate from SMPCs.^[35]^ However, the feature plot showed that the PEG3/PW1 gene is expressed in tumor cells (Fig. 2C). *PEG3*/*PW1* is a marker of a subset of vascular-related EPCs.^[36]^ Therefore, ERM in the pelvic may also originate from EPCs. Pseudo-time analysis is performed to explore the origin of ERM in the pelvic. The results show that cluster 12 comprises MSCs (*TAGLN*^hi^, Fig. 4A) and is mainly located at the left of the evolutionary trajectory (Fig. 4B), so the left side represents the start of the evolutionary trajectory. EPCs are derived from MSCs differentiation. If the tumor cells are derived from EPCs, cluster 13 should be located on the evolutionary trajectory between MSCs and tumor cells. However, cluster 13 is not located at that location (Fig. 4B). Therefore, ERM originates from SMPCs rather than EPCs.

In the evolutionary process of ERM, *TAGLN* gene is expressed briefly and it is prior to tumor evolution during early skeletal muscle differentiation (Fig. 4A).^[37]^ It can be used as evidence that ERM originated from SMPCs too. After that, MSCs are differentiated into CAFs and tumor cells, and the *VEGFA* gene is expressed in CAFs and tumor cells (Fig. 4A). Clusters 0 and 7 exhibit functions of sensing and adapting to oxygen changes and promoting angiogenesis. They are on the right branch of the evolutionary trajectory (Fig. 4A). Therefore, combined with the expression of the *VEGFA* gene in pseudo-time, we speculated that the *VEGFA* gene is the first group of genes expressed during tumorigenesis. At the end of tumor evolution, *MKI67*, *TOP2A*, and *CDK1* genes began to express (Fig. 4A). These genes are expressed in Clusters 2, 5, 8, and 9 and relate to the high proliferation of tumors, which are manifested in the increase of tumor malignancy. GO/KEGG analysis showed that cluster 11 is also highly proliferative, but the position on the evolutionary trajectory is different from clusters 2, 5, 8, and 9 (Fig. 4B). It may be related to the different growth rates during tumor growth. With the evolution of tumor cell subtypes, the differences between tumor cells and normal cells increase, and the growth rate of tumors increases. At the cellular level, the number of highly proliferative tumor cells also increases.^[38]^ Therefore, clusters 2, 5, 8, and 9 are mainly distributed at the end of the evolutionary trajectory, cluster 11 is distributed in the entire evolutionary trajectory. The number of highly proliferative tumor cells at the end of the evolutionary trajectory is higher than the tumor cells at the beginning. There are also some tumor cells that are expressed throughout the evolution of the tumor. There is no marker gene to define the remaining clusters, so GO/KEGG analysis is used to show the functions of every cluster. The epigenetic modification of cluster 6 is enhanced, and epigenetic modifications are altered throughout the whole process of tumor initiation and progression,^[39]^ so it is distributed throughout the evolutionary trajectory. The metabolism of nucleoside phosphoric acid is enhanced in clusters 4 and 10, and it is distributed at the middle of the evolutionary trajectory, this event should occur in the middle of the evolution of tumor cells. The DEGs of clusters 1and 3 are mainly involved in ossification and ossification regulation. Clusters 1and 3 are distributed throughout the evolutionary trajectory, but their role in tumor evolution is still unclear. The existing literature can only prove that ERM has an osseous component, namely, ossification can occur during the evolution of ERM.^[40]^ The evolutionary history of ERM is shown in Figure 3A, and GO analysis results were shown in Figure S1, Supplemental Digital Content.

By studying the heterogeneity in ERM, clinical diagnosis and treatment of ERM will also help. *Ki-67*, a marker of cell proliferation encoded by the *MKI67* gene, is often used to evaluate the degree of malignancy ERM. Clinical trials have shown that the lymphatic spread^[41]^ and prognosis^[42]^ of ERM are correlated with the *Ki-67* index. However, *Ki-67* has some limitations as a marker of cell proliferation. The *MKI67* gene is not expressed in the G1 phase, and in vitro cell culture experiments showed that *Ki-67* is not needed for mammalian cell proliferation.^[43]^ Our results demonstrate that clusters 3 and 12 are highly proliferative but do not express the *MKI67* gene (Fig. 1C). The question still lies in how to calibrate the *Ki-67* proliferation index. Feature plots show that *TOP2A* and *CDK1* genes are expressed in cluster 3 (Fig. 1C), which means that the classical *Ki-67* index may be corrected by the expression products of these 2 genes.

In conclusion, this paper revealed the cellular composition of ERM tumor tissue, revealed the heterogeneity in ERM, explored the evolutionary history of ERM, and improved the method of evaluating the degree of malignancy of ERM.

## Acknowledgments

We thank Xing-Yu Huo for editing the English text of a draft of this manuscript.

## Author contributions

**Conceptualization:** Bo Hong, Tian Xia, Rui Dong.

**Data curation:** Bo Hong, Tian Xia.

**Formal analysis:** Bo Hong, Tian Xia, Chun-Jing Ye, Yong Zhan, Ran Yang, Jia Liu, Yi Li, Zhi-Xue Chen.

**Funding acquisition:** Rui Dong.

**Investigation:** Bo Hong, Tian Xia.

**Methodology:** Bo Hong, Tian Xia, Rui Dong.

**Project administration:** Wei Yao, Kai Li, Jia Wang, Kui-Ran Dong, Rui Dong.

**Resources:** Wei Yao, Kai Li, Jia Wang, Kui-Ran Dong, Rui Dong.

**Software:** Bo Hong, Tian Xia, Chun-Jing Ye, Yong Zhan, Ran Yang, Jia Liu, Yi Li, Zhi-Xue Chen.

**Supervision:** Wei Yao, Kai Li, Jia Wang, Kui-Ran Dong, Rui Dong.

**Validation:** Bo Hong, Tian Xia.

**Visualization:** Bo Hong, Tian Xia.

**Writing – original draft:** Bo Hong, Tian Xia.

**Writing – review & editing:** Wei Yao, Kai Li, Jia Wang, Kui-Ran Dong, Rui Dong.

## Supplementary Material

Supplemental Digital Content

## Supplementary Material

Supplemental Digital Content
