# Supplementary figures and images for: Single-cell transcriptional profiling reveals the heterogeneity in embryonal rhabdomyosarcoma
Source: Medicine (Baltimore). 2021 Aug 6;100(31):e26775. doi: 10.1097/MD.0000000000026775 (PMC8341243; doi:10.1097/MD.0000000000026775)

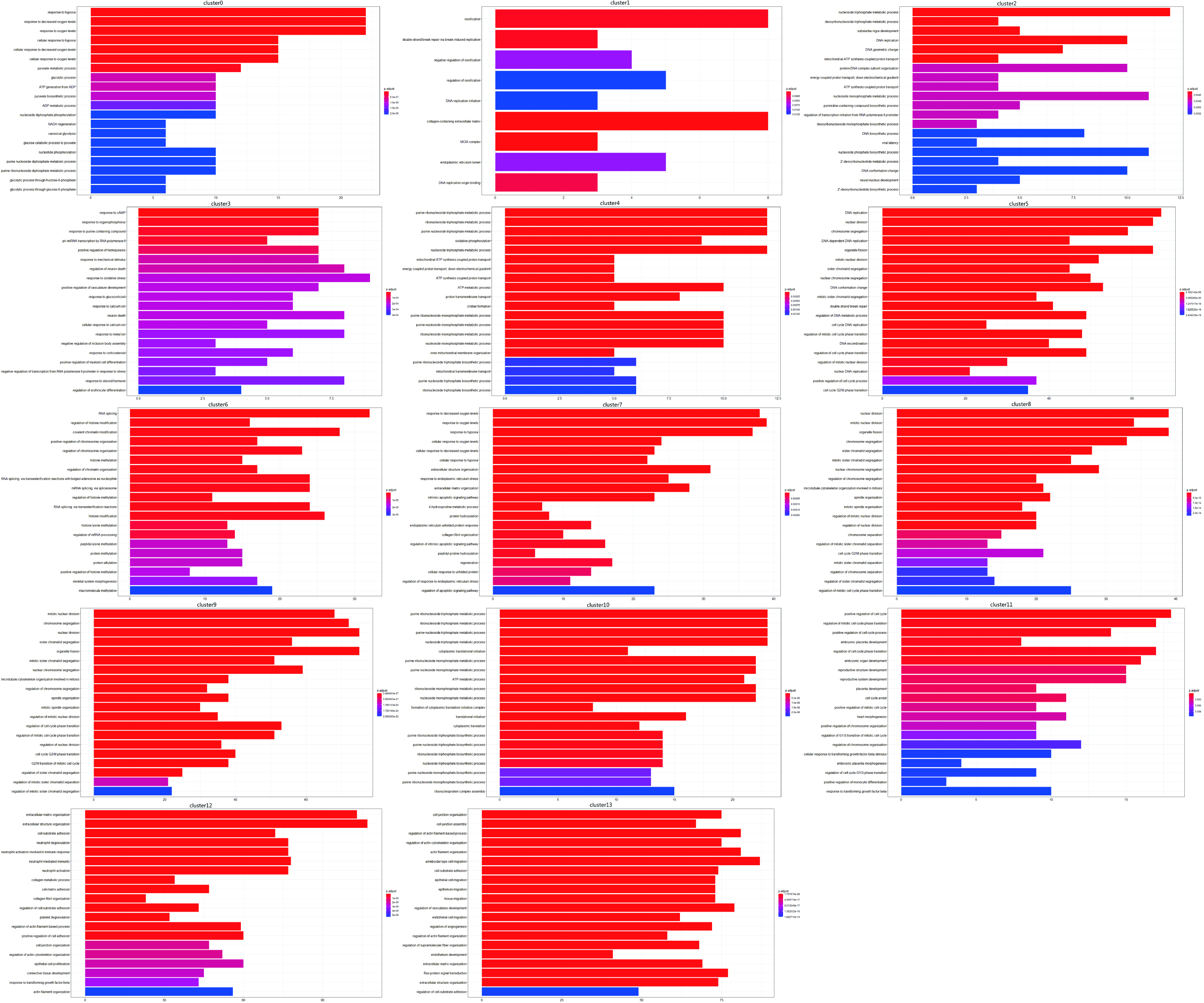

Supplement: Supplemental Digital Content [file medi-100-e26775-s002.jpg]
